# Semantic algorithms can detect how media language shapes survey responses in organizational behaviour

**DOI:** 10.1371/journal.pone.0207643

**Published:** 2018-12-05

**Authors:** Jan Ketil Arnulf, Kai Rune Larsen, Øyvind Lund Martinsen

**Affiliations:** 1 Department of Organizational Management and Leadership, BI Norwegian Business School, Oslo, Norway; 2 Leeds Business School, University of Colorado at Boulder, Boulder, Colorado, United States of America; The University of Memphis, UNITED STATES

## Abstract

Research on sensemaking in organisations and on linguistic relativity suggests that speakers of the same language may use this language in different ways to construct social realities at work. We apply a semantic theory of survey response (STSR) to explore such differences in quantitative survey research. Using text analysis algorithms, we have studied how language from three media domains–the business press, PR Newswire and general newspapers–has differential explanatory value for analysing survey responses in leadership research. We projected well-known surveys measuring leadership, motivation and outcomes into large text samples from these three media domains significantly different impacts on survey responses. Business press language was best in explaining leadership-related items, PR language best at explaining organizational results and “ordinary” newspaper language seemed to explain the relationship among motivation items. These findings shed light on how different public arenas construct organizational realities in different ways, and how these differences have consequences on methodology in research on leadership.

## Introduction

Where–and how–does the language we use to describe leadership and organizations emerge? Language is neither given by nature nor static, but reflect continuous cultural developments [1–3]. Language is the primary tool by which humans construct social realities [4–6]. This is not only true for the everyday language of ordinary people, but also for communities of professional practitioners such as scientists [7–10] and managers [11–15].

Public media probably serve as an important arena for constructing leadership phenomena in language, which have been shown to exert powerful influence of constructing leaders as heroes or failures [16–19]. Language related to leadership is probably also shaped by managers themselves through communities of practice [20,21] since rhetorical skills are important in leadership practice [21–24]. A third important community of language users are employees or followers, who may hold different ideas about leadership from their superiors but who also need to engage in sensemaking about leadership as they are affected by it [25–30].

The purpose of this study is to explore how the language used in public media may be related to responses to leadership measurement instruments. Using digital methods of text analysis, we build on the recently proposed semantic theory of survey response (STSR), showing how semantic properties of survey items seem embedded in the ensuing statistical patterns [31–34]. By applying the methods suggested in STSR to a broad sample of respondents and leadership questionnaires, we hope to make three contributions: The first is to open a quantitative approach to study the social construction of leadership in the media applying the recent advances in digital text analysis. To our knowledge, this is a new field of study. Secondly, since surveys are a predominant research method in OB generally and in leadership in particular [35,36], we believe it is important to expand our knowledge about sources of influence on to better control and interpret measurement models [37–39]. Thirdly, we want to contribute to expand our understanding of STSR itself. As recent publications show, the semantic influences on survey statistics are not just substantial but may actually replace the measures of attitude strength completely [31,34,40]. It is therefore important to explore the importance of contextual factors in semantic influences as text algorithms are not always sensitive to these [41].

## Theory

### Constructs as semantics instead of attitude strength

The semantic theory of survey response (STSR) has shed new light on an old discussion in social science, the nature of latent constructs [42–45]. The most frequent tool in measurement and validation of latent construct remains the Likert-type scales asking people to rate their attitude strength [36,46,47]. The measurement of the latent construct properties is assumed to be linked to the attitude strength, that is, the intensity by which the respondents react to the subject matter of the survey. The recent studies in STSR question this assumption by showing that the measures of attitude strength may actually be filtered out as noise if the structure of the survey instrument is sufficiently dependent on semantic properties of the items [34]. When this happens, the quantitative patterns in the statistics are no longer indicative of attitude strength, but rather an expression of the semantic linkage between the items. Not only items, but whole constructs may have overlapping meanings, making not only survey statistics semantically explainable [40,48] but also explain why constructs overlap in meaning despite having different names, the so-called jingle/jangle-problem [49].

The shift from attitude strength to semantic networks is a return to the original meaning of the word “construct” as precisely a logical construction [44,50]. Concepts such as “leadership” and “motivation” are probably not objectively given entities but socio-cultural constructions in language [4–6,51–54]. Even the term “organizational performance” is a social construction of vague delineation [55,56].

Our use of digital algorithms in STSR makes it possible to bridge the quantitative studies of organizational behaviour with the social-constructionist approach, two strains of scholarship that have hitherto mostly been separate domains [57].

In the following, we will outline the social emergence of “leadership”, “motivation” and “results” as three important constructs in management science, and argue that our text algorithms allow us to trace how these constructs are operationalized in surveys depending on language from different media domains.

### Sensemaking as emergence of concepts and constructs

Language develops along with culture, politics and technology, although the precise reasons for and driving factors in this development are still debated [1,9,58,59]. On a macro level, societies seem to develop linguistic structures to deal with challenges in their habitat, a phenomenon that in its most extreme version has been termed the “linguistic relativity hypothesis” [60,61]. While the details have been debated [62], it seems unquestionable that language does evolve as gradual adaption to socio-economic changes [2,6,63–65] in a way called “thinking for speaking” [66].

On an organizational level, linguistic evolution has gained importance for theory and practice in organizational behaviour through theories on “sensemaking[11–13,21,67]. Sensemaking may be defined [68] as the “ongoing retrospective development of plausible images that rationalize what people are doing”, resulting in a “consensually constructed, coordinated system of action” (p. 40). Subjects will attend to problems and important experiences, capturing and condensing them in language [69]. A community of speakers will therefore adopt linguistic habit central to their challenges, evolving concepts to communicate about common problems, or in the words of March, “people say “Yell if you’re in trouble”, not “Yell as long as you are OK” [70].

Professionally or organisationally relevant experiences become embedded in the linguistic habits of organisational actors. Weick [69] shows how organisational actors identify non-verbal “hunches” in the ongoing flux of activities, then label these “hunches” to make them accessible in communication, and hence manageable. Underscoring the emergent and creative quality of this process, he describes people who are able to do this as “change poets”.

**Fig 1:**
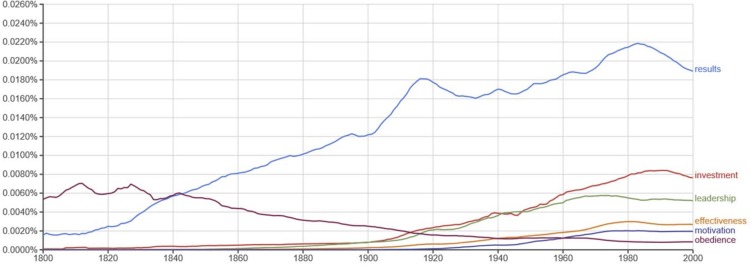
The historical development of the words “results”, “investment”, “leadership”, “effectiveness”, “motivation” and “obedience” in English during the years 1800–2000 (source: Google Ngram Viewer).

Sensemaking contributes to linguistic innovation at both macro and micro levels in society. At the macro level, we find concepts used in Organisational Behaviour (OB) such as “leadership”, “motivation” and organisational outcomes or “results”. It is possible to use publically available databases such as Google nGram (https://books.google.com/ngrams) to explore the historical emergence and popularity of words and terms in the world’s major languages, as we exemplify in Fig 1. For the present purpose, we have traced the emergence of organizationally relevant words such as “leadership”, “motivation” and “results” in all printed words of American English during the years 1800–2000. To illustrate the conceptual context, we have added the two concepts “investments” and “effectiveness”, as concomitants to an interest in “results”.

The word “results” emerged with increasing importance during the industrial revolution, expressing an interest in the relationship between employed capital, technologies and industrial productivity [71]. This co-emerged with the shareholding company with limited liability was as a permanent legal subject in Great Britain in 1844 [72]. Almost immediately adopted in the US, this way of organising capital and work expanded quickly and gave rise to the US-style business school, where the word “leadership” developed as a preferred concept for explanations of organisational performance [73–75]. The concept of “motivation” appeared later, possibly as the specifically human counterpart to “effectiveness” as returns on investments in technical innovations. The decline of the term “obedience” in Fig 1 contrasts the transition from traditional compliance to motivation-oriented leadership in today’s management discourse [76].

Similar emergent cultural properties of language have recently been explored in other digital text algorithms [65,77]. We use Google nGram illustrate how central OB concepts emerged on a macro level from the sensemaking needs of different stakeholders in the economy, socially constructing the work of shareholders (“investments” and “results”), managers (“leadership”), and employees (“motivation”) [4]. Together, the words “result-focused”, “leadership” and “motivation” may be the most frequently used terms to describe and rate the capabilities of business school graduates [73].

When such concepts are taken to be “constructs” by researchers who subject them to construct validation, they must be transformed into survey items that serve as operationalizations of the construct [42,78,79]. Overviews of this practice suggests that researchers invest much effort in making sure that the psychometric structures of such survey items are also aligned with face validity and other conceptual work on the scales [80]. Based on STSR, we claim that this work is actually a translation of the linguistic properties of the focus concepts into a series of statements (survey items) that show if the respondents agree to the coherence of these sentences. However, the semantic properties of the items will reflect their heritage from the social arenas that created them, which is what we intend to explore using the semantic algorithms.

### STSR and semantic space

The semantic theory of survey response (STSR) was established by applying text algorithms to analyse how the wording of survey items may shape the survey statistics [31,33,34,81]. Various kinds of semantic algorithms are available and being used for research and commercial text handling purposes [e.g., 41,77,82,83–86]. The common purpose of the algorithms we use here is to measure quantitatively how two or more texts overlap in meaning.

The present research approach exploits some important differences between two types of these algorithms. The first type is Latent Semantic Analysis (LSA), which derives its data about meaning from analysis of existing bodies of texts, so-called semantic spaces. For our purpose here, one may think of LSA as “learning” language from exposure to naturally occurring language, much in the same way that children do [87]. The other approach we use is termed MI [86,88] and derives its knowledge from a lexical database of words and definitions such as WordNet [89–91]. It is not sensitive to context- and subgroup-specific knowledge about language. In contrast, LSA will pick up meaning from the text fed into it. If this text has localized properties, it may be discernible in meaning as “learnt” by LSA.

By comparing the output of LSA from various media domains with the purported “lexical” output of MI, we can explore if there are meaningful linguistic differences between these domains, and in turn explore if these differences have consequences on responses to leadership surveys. A few more explanations of these algorithms are therefore necessary.

LSA can be seen as a mathematically based theory of meaning [84,92] in that it “demonstrates a computational method by which a major component of language learning and use can be achieved” [84]. LSA functions by analysing texts to create a high-dimensional “semantic space” in which all terms have specific locations, represented as vectors [81]. The algorithm “understands” new texts as combinations of term (word) vectors in this space. LSA aggregates the word contexts in which a given word does/does not appear and provides a set of constraints that determines the similarity of meanings of words and sets of words. Thus, when two terms occur in contexts of similar meaning–even in cases where they never occur in the same passage–the reduced-dimension solution represents them as similar. We use this representation to compare similarities in survey items [33,83,93].

Because LSA derives meaning directly from a defined semantic space (such as a selected collection of books, articles, or similar), the composition of any semantic space determines what LSA “learns”. This is comparable to how co-workers embedded in the same “community of practice” may adopt specific vocabularies [20,94]. We want to apply the same LSA algorithm to different semantic spaces composed of different and mutually exclusive media domains. The interesting question is whether these yield different representations of OB-related concepts.

MI differs from LSA in that its values reflect only lexical knowledge encoded by a team of linguists between 1990 and 2007, for a set of 147,278 unique words existing in the WordNet database [86,88]. The MI algorithm first identifies single words in each sentence and computes similarity as the shortest distance between these words’ synsets (sets of synonymous words) in the WordNet hierarchy. Word-similarity scores are then used as inputs in a formula computing sentence-level similarity. Therefore, MI output builds first on the foundation of word-level meaning and then on the sentence-level structures where the words appear. MI “knows” nothing about special terms used by professional communities whereas LSA can target specific semantic spaces belonging to defined groups of speakers. Together, they may cover multiple aspects of actual language usage. To spare the reader of details, we describe details of the algorithms in Appendix A.

By using the lexical MI values as control variable for the domain-specific LSA cosines, we show how the LSA residuals represent differences in language usage between different media domains. We can then link the language of these media domains to the concepts of leadership, motivation and outcomes by showing differences in their explanatory value for survey responses on corresponding measurement scales.

The output of these algorithms is cosines for LSA and a range of fractional values for MI ranging between 0 and 1, henceforth referred to collectively as semantic similarity values. Higher numbers indicate higher semantic similarity between the two items. LSA cosines can take negative values and bear some similarity to correlation coefficients; MI’s semantic similarity scores are not cosines and do not produce negative values at all. We describe the handling of this problem in the methods section.

**Fig 2:**
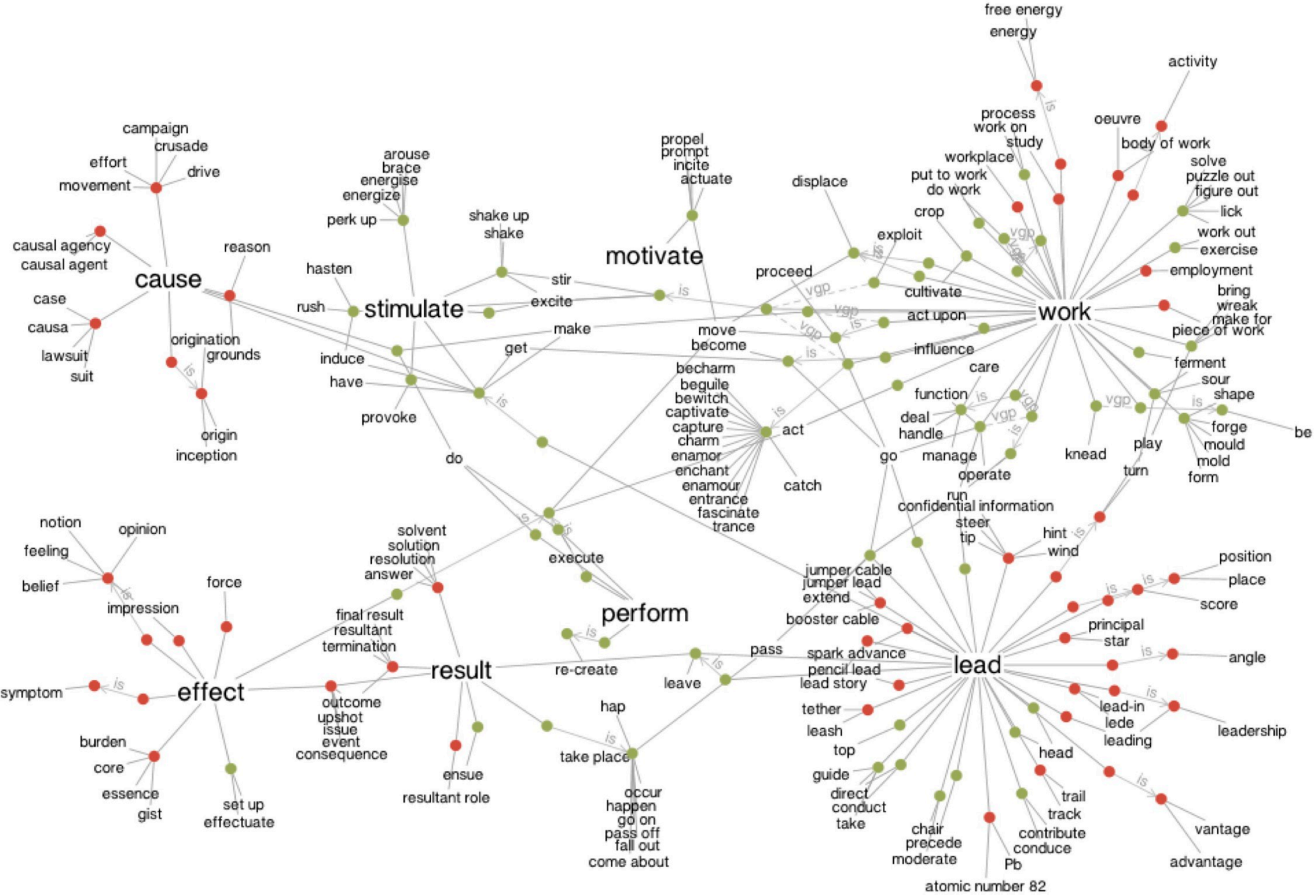
The semantic network of the concepts “cause”, “stimulate”, “work”, “lead”, “perform”, “result” and “effect” in WordNet (Source: http://www.WordVis.com). For our analysis, this means that we treat the observed survey responses in the traditional sense as quantitative measurements, and we apply statistical regression techniques to map semantic indices on these survey responses. It is also possible in one sense to separate semantic measures by using other variables as control variables. We do this to demonstrate that the semantic characteristics of surveys are related to the measurements of attitudes and their basic statistical properties. At some point, however, linear or even cubic regression models will not be appropriate for the separation of variables that originate from a 300-dimensional space, such as LSA [84,87,99].

Another comment about the quantitative nature of LSA values is necessary. For measurements of attitudes by Likert scales, it is commonly assumed that the underlying measurements are unidimensional. A single attitude such as that towards leaders or one’s own work is assumed to be quantifiable as more or less in favour of a statement [95–98]. LSA values, however, are not quantifications of assumed uni-dimensional structures in the same sense. The LSA cosine or MI value of any item pair can be interpreted as the degree to which or likelihood with which people will read similar meanings into these items, as words and propositions hold multiple linkages in semantic networks. Fig 2 is a visualisation from WordVis, a graphic representation of the WordNet database (http://www.wordvis.com), to show a two-dimensional representation of the multiple relationships in a semantic network.

### Hypotheses about linguistic relativity in surveys

What we have argued so far is that: a) central concepts in OB are semantic constructions; b) these semantic constructions are subject to sensemaking and linguistic relativity; c) survey responses are dependent on the semantic habits of the respondents; and that d) semantic text algorithms may be differentially sensitive to language as used in different types of social discourse. Based on these premises, we will now deduct our guiding hypotheses.

Outcomes, or “results”, are general terms that may be of interest to a wide community. A reputation built on achievements and result-orientation is generally good for business and one of the main purposes of corporate communication [55,100]. Business stakeholders and the business press have in recent decades developed a tough “show me the money” type of attitude that has affected corporate governance and created increased pressure on managers and business leaders to perform [56,74,101].

**H**_**1**_: LSA semantic similarity indices generated from a semantic space created from *PR Newswire* will show the strongest explanatory value for survey items measuring organisational outcomes.

According to Khurana’s analysis of the history of business schools [73], the word “leadership” has become synonymous with success in business. This form of rhetoric seems strengthened by recent publications with titles suggesting leadership excellence, such as “the world’s most successful leaders” [75]. The interest in leadership is linked to an interest in predicting later organizational success [56,102]. Descriptions of leadership are frequently conducive to heroic, exaggerated claims that may sometimes work as self-fulfilling prophesies, but may also collapse in disappointment or downright fraud [16,18,33,103,104]. The business press could therefore be particularly interested in leadership as a concept that helps make sense of and predict organizational performance.

**H**_**2**_: LSA semantic similarity indices generated from a semantic space created from business press texts will show the strongest explanatory value for survey items measuring leadership.

Motivation, on the other hand, could be more strongly related to the subjective views of employees, precisely for the reason that the motives of employees may not be united with those of the owners or the top managers [105]. Contrasting views on work motivation can be seen as different ways of constructing the interests of employees and organisations in mutual cooperation [106–109]. It is therefore necessary that survey items concerning motivation are closely linked to the common language of most people. Questions meant to elicit motivational statements from employees are possibly more tightly related to language in the general press than to the language of *PR Newswire* or the business press.

**H**_**3**_: LSA semantic similarity indices generated from a semantic space created from ordinary newspaper texts will show the strongest explanatory value for survey items measuring motivation.

## Methods

### Measures

The dataset in this study are a re-analysis of the three subsets of data covering leadership items previously used in the 2014 article by Arnulf & al. We believe this is justifiable because the analysis presented here is completely different, and the readers can consult the previous study to gain a complementary understanding of how these data fit more traditional psychometric criteria. The semantic measures will never change unless the algorithms are changed, and the way we use them here is not covered by the previous study.

Another feature of the present analysis is that we do not group the analysis according to the respondent samples. Instead, the focus here is on the samples of *item pairs* from a broad range of scales. We are grouping the item texts into our categories of interest–leadership, motivation, and outcomes–and analyse their semantic predictability as being responded to across samples of respondents.

As measures of leadership, we chose the 36 first items describing leadership behaviours from the Multifactor Leadership Questionnaire (MLQ) published by Avolio, Bass and Jung [110]; seven items measuring leader–member exchange, or LMX [111]; the Ohio State University two-factor theory of leadership was measured by the Leader Behaviour Description Questionnaire (LBDQ) [112], from which we included 10 items measuring initiation of structure and 10 items measuring consideration.

Measuring motivation, we used seven items measuring economic-exchange perceptions and eight items measuring social-exchange perceptions (developed and validated by Shore, Tetrick, Lynch, & Barksdale) [113]; six items measuring intrinsic motivation (developed and published by Kuvaas) [114]; eight items of affective organisational commitment published by Meyer, Allen and Smith [115]; and three items measuring job satisfaction published by Cammann, Fichman, Jenkins and Klesh [116].

As outcome variables, we included the last nine outcome measures from the MLQ that are usually taken as indicators of outcomes instead of leadership behaviors [110,117]; seven items measuring organisational citizenship behaviour (OCB) validated by Van Dyne and LePine [118]; turnover intention (TI) measured by five items also published by Kuvaas [119]; and self-rated work quality and work effort, each measured by five items published by Kuvaas [114].

In total, there were 88 items, producing a matrix of 3,828 unique pairs of inter-item correlations. For all item pairs, we obtained parallel sets of semantic indices applying MI, as well as three sets of LSA cosines from respectively (1) *PR Newswire*, (2) business press periodicals and (3) common newspapers.

### The respondent samples

This dataset consisted of semantic similarity indices and observed correlations from a number of scales, but used in three different samples of respondents. The first sample contained 1,649 respondents from various units of a financial company that all of them responded to all 45 items in the MLQ. For 424 (25%) there were no demographic characteristics available. Among those whose background was known, 51.1% were males and the average age was 46 years (SD = 11 years). Two percent belonged to top management, 26% were middle managers and 71% did not hold management positions. All participants rated their immediate supervisor. The survey was conducted in Norway, using a Norwegian translation of the MLQ. This was originally selected for convenience and for the purported cross-language functionality of LSA [120–123].

The second sample consisted of 255 employees at a governmental research agency, mostly scientists and engineers. Of these, 66.7% were male and the mean age was 38 years. Twenty-five percent rated themselves as managers and the rest termed themselves as “project team members”. Their mean tenure was 7.5 years. They filled out an online questionnaire covering only the 36 leadership items of the MLQ as well as the items gauging economic and social exchange, intrinsic motivation, TI, OCB, perceived work effort and perceived work quality.

The third sample consisted of 981 civilian and military employees in the Royal Norwegian Armed Forces. The precise demographics are restricted information for this dataset, but the survey was requested by the Norwegian Ministry of Defence for internal purpose, and the demographics therefore reflect a representative sample of full-time employees and a majority of males with a mean age of around 35. Most of the subjects are likely to have personal leadership experience, being officers of various ranks. These responded to a combination of items measuring transformational leadership from the MLQ, the seven LMX items, the 20 items from the LBDQ, affective commitment, OCB and turnover intention.

From each sample, we collected a set of data consisting of all surveyed inter-item correlations with their respective MI and LSA values. Note that some item pairs appear in all datasets, such as the transformational leadership items in the MLQ. Others only appear in one of the sets, such as LMX, OCB and the job satisfaction items. The first sample of respondents yielded information from 990 item pairs (responding to the 45 item version of the MLQ), the second sample 2,346 item pairs and the third sample 3,081 item pairs (in total 6,417 pairs). Note that these were the numbers of item pairs sampled from the respondents. As we group our item pairs according to the item pair types instead of respondent samples, the numbers of items within each group depends on the items included in each category (leadership, motivation, or results).

In order not to confound linguistic effects between the scales, we used only the item pairs that belonged to the same domain, i.e. leadership-leadership, motivation-motivation and results-results, which further reduced the number of item pairs to 2,860 pairs of item correlations. Some inter-item relationships were tested in more than one subset of the samples, as their empirical correlations may have been different in the various samples. The total sample of surveyed individuals was 2,885 persons. We want to emphasize that the sample size in the present study is not the number of respondents in the empirical surveys, but the number of inter-item relationships that can be mapped onto a semantic structure together with an observed correlation from the respondents.

### Analytic procedures

Our main methodological aim was to explore whether cosines derived from three semantic spaces would be significantly different from each other and have significantly different explanatory value for the theoretically predicted parts of our surveys. There are many vector-focused similarity algorithms available, but we used LSA because Larsen and Bong [49] found that for survey items, LSA clearly outperformed LDA. We also chose LSA because of its simplicity and wide acceptance (> 13,000 Google Scholar citations), and because we want to leave no doubt about the basic nature of the phenomena underlying STSR.

Our first step was to establish the meaning of the survey items though LSA detection of accumulated knowledge and semantic relationships within relevant texts. As theoretically hypothesised above, we assumed business language to be most strongly represented by the common business press. Leadership language was assumed to be most saliently represented by PR-related texts, and general newspaper texts would represent text used by the average reader. Semantic spaces were developed accordingly.

The business-press texts were excerpts from *The Wall Street Journal*, *Business Week*, *Forbes* and *Fortune*. These excerpts covered a total of 84,836 texts from the years 1998–2007, covering a total of 45,816,686 words and 169,235 unique words.

The PR statements were taken from *PR Newswire*, covering the years 2003–2007. This sample included 216,963 texts with 151,450,055 total words and 423,001 unique words.

The news excerpts were from *The New York Times*, *Los Angeles Times*, *Chicago Tribune*, *The Washington Post*, *The Boston Globe*, *USA Today*, *Houston Chronicle*, *San Francisco Chronicle* and *The Denver Post*. The years covered were again 1998–2007, including 162,929 texts with 107,239,064 total words and 286,312 unique words.

Every survey item in the study was projected into each semantic space to generate its mathematical representation (vector). These representations were, in turn, compared, allowing computation of cosine angles between all the item vectors, with higher cosines indicating higher similarity between items. This procedure was repeated for all three semantic spaces. LSA establishes the meaning of an expression by mapping its usage in each semantic space. The output is a cosine value indicating how similar their usage appears in semantic space [124,125].

LSA’s language “understanding” in the three semantic spaces probably has a strong common lexical basis. To enhance the differences among the semantic spaces, we treated the semantic similarity indices from the MI algorithm as a control variable.

As described above, MI values do not take negative signs, and only a few LSA values are negative as the meaning of this is not equal to negative correlations. There are two strategies for dealing with the sign issue. It is possible to remove all signs and use absolute semantic similarity score values and absolute survey response patterns, but this also greatly reduces the information in the data.

The second approach is use of a content-based approach to align the signs. Backward-scored items are easily corrected by content. However, the MLQ does not contain such scores while 264 (26.7%) of 990 pairs of items are negatively correlated. Theory suggests that two scales, ‘laissez-faire’ and ‘passive management by exception’, are likely to relate negatively to effective leadership. Common sense indicates that pairing items from these scales with items from other scales would correlate negatively, such as items stating that: a) a manager is unapproachable when needed; and b) that the same person uses appropriate methods of leadership. The surveyed responses to these items correlate −.42 in our sample, and the semantic identity values range between .38 and .75. Based on this, we allowed the signs of semantic identity scores to be negative for all pairs of items from ‘laissez-faire’ and ‘passive management by exception’ (except for pairs of items within these scales), using theoretical knowledge available before beginning the empirical survey (correctly identifying 255 of the 264 negative correlations, p = .000). In the case of non-identified items the signs were kept unchanged, so that the semantic values will predict these poorly.

The steps involved in LSA and MI computation are described more precisely in Appendix A, and descriptions about detailed methodology used here is found in more detail in recent publications on the same matter [49,81,82].

As an additional check, we have re-analysed the findings while varying the numbers of dimensions in the semantic spaces. The outputs of LSA will be sensitive to two conditions that are not in themselves the focus of this study: The different sizes of the semantic spaces and the numbers of dimensions (k) used in the singular value decomposition (SVD). For this reason, we will also generate the LSA cosines for each semantic space using 2–500 dimensions to see whether the findings hold across dimensions. As we generate a range of estimates we can also explore their relationship with the size of each semantic space.

## Results

Table 1 shows the zero-order correlations among all variables in the study. The MI values and semantic cosines show strong mutual correlations, and correlate .55–.72 with the observed survey responses. Of the semantic similarity indices, the MI values show stronger correlations with the survey responses than the LSA cosines. Hence, to control for the common lexical basis, we regressed the cosines from the three LSA domains on the MI values and retained the residuals. As expected, the LSA residuals were no longer correlated with MI, only faintly correlated with the observed survey responses, and their mutual correlations were also reduced. In the third set of variables, we also controlled the LSA cosines for the respective two other LSA domains. These LSA residuals were now not mutually correlated at all, but it is interesting to see that their correlations with observed survey variables actually increased in magnitude. The values in Table 1 support our claim that MI values constitute a common lexical basis for all the semantic similarity indices, and that there exists unique variation within each separate set of LSA cosines.

**Table 1:** Zero order Spearman correlation among all variables in the study (* p < .050, ** p < .001).

Correlations (N = 2,860)	1	2	3	4	5	6	7	8	9	10
1. MI values										
2. BIZ cosines	.83**									
3. NEWS cosines	.82**	.96**								
4. PR cosines	.84**	.97**	.94**							
5. BIZ cos. controlled for MI	.00	.56**	.50**	.49**						
6. NEWS cos. controlled for MI	.00	.49**	.58**	.44**	.87**					
7. PR cos. controlled for MI	.00	.50**	.46**	.55**	.89**	.80**				
8. BIZ controlled for MI, NEWS and PR	.00	.21**	.00	.00	.37**	.00	.00			
9. NEWS controlled for MI, BIZ and PR	.00	.00	.28**	.00	.00	.49**	.00	-.58**		
10. PR controlled for MI, BIZ and NEWS	.00	.00	.00	.25**	.00	.00	.45**	-.65**	-.12**	
11. Survey correlations from respondents	.80**	.62**	.61**	.63**	-.08**	-.09**	-.08**	.01	-.04*	-.01

We then proceeded to test the three hypotheses in various ways. The most straightforward way was to split the data by item domains (leadership, motivation, results) and perform a hierarchical regression with survey responses as the dependent variable and MI values in the first block. When entering the three types of LSA cosines in the second block, the ensuing patterns of standardised betas indicated their mutual importance. As can be seen in Table 2, PR-generated semantics were significantly related to the outcome-measuring items, and LSA data from other semantic spaces showed less explanatory value. This supports **H**_**1**_, which stated that the semantic similarity indices generated from *PR Newswire* would be strongest related to items measuring organisational outcomes.

**Table 2:** Stepwise hierarchical regression with respondent survey correlations as dependent variable, by item domains (p for adj. R^2^ increase in step 2 < .01 for every model. * p < .050, ** p < .001).

Item domain			Standardised β	t	Df	F	Adj. R^2^
Leadership	Step 1	(Constant)		24.54**	2,248	4,419.53	.66
		MI values	.81	66.48**			
	Step 2	(Constant)		22.46**	2,245	1,114.73	.67
		MI values	.85	43.45**			
		BIZ cosines	−.18	−3.37**			
		NEWS cos.	.04	1.02			
		PR cosines	.10	2.47*			
Motivation	Step 1	(Constant)		5.14**	244	328.40	.57
		MI values	.76	18.12**			
	Step 2	(Constant)		5.48**	241	106.79	.63
		MI values	1.45	8.23**			
		BIZ cosines	.02	0.05			
		NEWS cos.	−1.23	−4.05**			
		PR cosines	.48	1.23			
Results (outcomes)	Step 1	(Constant)		23.66**	362	1,249.91	.78
		MI values	.88	35.35**			
	Step 2	(Constant)		22.66**	359	344.02	.79
		MI values	.31	2.73**			
		BIZ cosines	−.26	−1.24			
		NEWS cos.	.10	0.81			
		PR cosines	.75	3.85**			
Total sample	Step 1	(Constant)		32.93**	28,268	5,153.17	.64
		MI values	.80	71.79**			
	Step 2	(Constant)		30.05**	2,855	1,329.82	.65
		MI values	.93	45.14**			
		BIZ cosines	.03	.50			
		NEWS cos.	−.14	−3.51**			
		PR cosines	−.04	−0.98			

For the leadership-related survey responses, the effects of the BIZ-related (business press) cosines were the strongest indicator among the LSA values. The PR cosines were also significantly related to survey responses in the leadership domain but not as strongly, and the NEWS cosines did not show up as significant in the analysis. This supports **H**_**2**_, which proposed that the semantic similarity indices from business press texts would be most strongly related to items measuring leadership.

And finally, **H**_**3**_ stated that LSA cosines from ordinary newspaper texts would show the strongest explanatory value for survey items measuring motivation. Again, this hypothesis seems supported in that these LSA cosines were indeed significantly and most strongly related to the motivation-related items, while the other two semantic domains did not contribute with significant variance.

We further tried to clarify the picture by performing two more regressions of the same kind as in Table 2, but this time using the residuals after controlling for MI and the other LSA cosines, respectively (variables 5–10 in Table 1). As can be seen from Table 3, these residuals still contain explanatory information (all models showing statistically significant R^2^ values) but the patterns were increasingly difficult to interpret. The reason for this is, as explained in the theory section above, that the mutual relationships among the semantic values are not uni-dimensional. In fact, the residuals are showing signs of curvilinear relationships. In the case of residuals, after controlling for both MI and the other LSA cosines, all quadratic equation models have higher R^2^ values than the linear functions.

**Table 3:** Multiple regression with respondent survey correlations as dependent variable, using “purified” LSA values by item domains.

		Controlled only for MI		Controlled for MI *and* the two other LSA domains		Fit statistics (both models)		
Item domain	Semantic indices	Standardised β	t	Standardised β	t	Df	F	Adj. R^2^
Leadership	(Constant)		42.06		42.06	2,246	124.65	.14
	BIZ residuals	.84	17.79	−.10	−1.96			
	NEWS residuals	−.42	−10.97	−.39	−8.40			
	PR residuals	−.55	−14.17	−.46	−8.87			
Motivation	(Constant)		3.25		3.25	242	39.02	.33
	BIZ residuals	−1.35	−7.49	.27	2.43			
	NEWS residuals	0.56	3.76	.61	6.58			
	PR residuals	1.17	6.54	.61	6.78			
Results	(Constant)		9.74		9.74	360	129.56	.52
	BIZ residuals	.52	5.145	1.78	19.14			
	NEWS residuals	−.09	−1.49	1.58	15.64			
	PR residuals	.29	3.39	1.49	16.77			

Interpreting and partialling linear regression weights is problematic because of the possible multicollinearities among the variables. This is almost certainly an issue here, where the semantic values are computed by related algorithms that are working on identical inputs (the survey items). To control for multicollinearity in the partialling of variable regression weights, we apply the techniques developed as a package in R by Nimon and Oswald [126]. They suggest a number of parameters to establish the true mutual impact of variables in the model, out of which we have chosen to report three: The first two are the *unique* and *common* effects as results of a commonality analysis, showing the unique contribution and their commonalities with the other variables. The third is the *conditional dominance*, where “conditional dominance weights have the potential to illuminate the properties of model predictors that can get lost in commonality coefficients” [126]. These calculations are presented in Table 4. It can be seen that the MI algorithm is generally the most influential source of data in a multiple regression with the empirical correlations as dependent variable, while all of them share large commonalities. However, for each of the item domains–leadership, motivation, and results, the hypothesized algorithms do appear as the most important predictor after MI. This means that the BIZ cosines were most important in explaining leadership, the NEWS cosines explain motivation, and in the case of results, the LSA cosines from the PRNewswire semantic space even dominate above the MI values. This analysis supports the conclusions from the hierarchical regression above.

**Table 4:** Uniqueness, commonalities and conditional dominance for all algorithms in the three subsets of items.

	Leadership items weights				Motivation items weights				Outcome items weights			
	*Beta*	*Unique*	*Common*	*Cond*. *Dom*.*3*	*Beta*	*Unique*	*Common*	*Cond*. *Dom*.*3*	*Beta*	*Unique*	*Common*	*Cond*. *Dom*.*3*
MI algorithm	.845	.282	.381	.282	1.449	.101	.472	.101	.318	.005	.772	.005
LSA BIZ	**-.177**	**.002**	.361	**.002**	.020	.000	.409	.000	-.154	.000	.772	.000
LSA NEWS	.038	.000	.348	.000	**-1.227**	**.025**	.400	**.025**	.050	.000	.750	.000
LSA PR	.100	.001	.375	.001	.481	.002	.466	.002	**.682**	**.007**	**.781**	**.007**
	Leadership items ranks				Motivation items ranks				Outcome items ranks			
	*Beta*	*Unique*	*Common*	*Cond*. *Dom*.*3*	*Beta*	*Unique*	*Common*	*Cond*. *Dom*.*3*	*Beta*	*Unique*	*Common*	*Cond*. *Dom*.*3*
MI algorithm	1	1	1	1	1	1	1	1	2	2	2	2
LSA BIZ	**2**	**2**	3	**2**	4	4	3	4	3	3	3	3
LSA NEWS	4	4	4	4	**2**	**2**	4	**2**	4	4	4	4
LSA PR	3	3	2	3	3	3	2	3	**1**	**1**	**1**	**1**

We then proceeded to explore the influence of the number of dimensions used in the analysis. The LSA cosines used in the calculations above were all generated with 300 dimensions, which is generally taken as an optimal number for *k* in LSA [81,127]. This will however depend on the semantic space and the purpose of the analysis and is therefore prone to variations. To explore the optimal number of dimensions for each semantic space, we generated the LSA cosines for all item pairs in sequence, using the whole range from 2 to 500 dimensions, then comparing how each output related to the empirically observed correlations.

**Fig 3:**
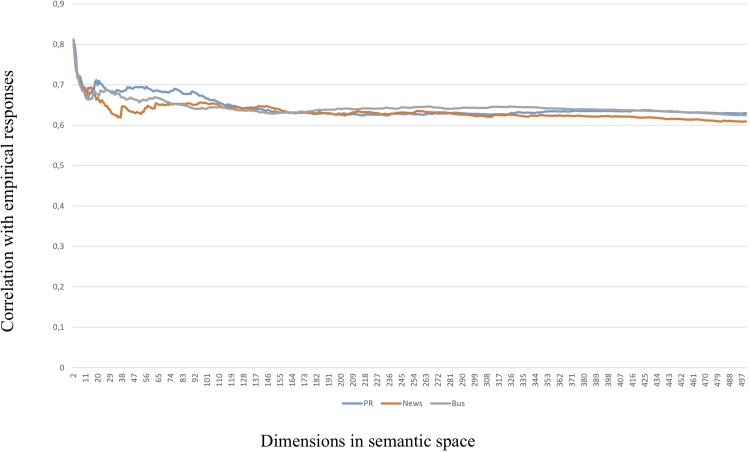
Correlations with whole dataset by semantic space and dimensions, 2–500 dimensions.

**Fig 4:**
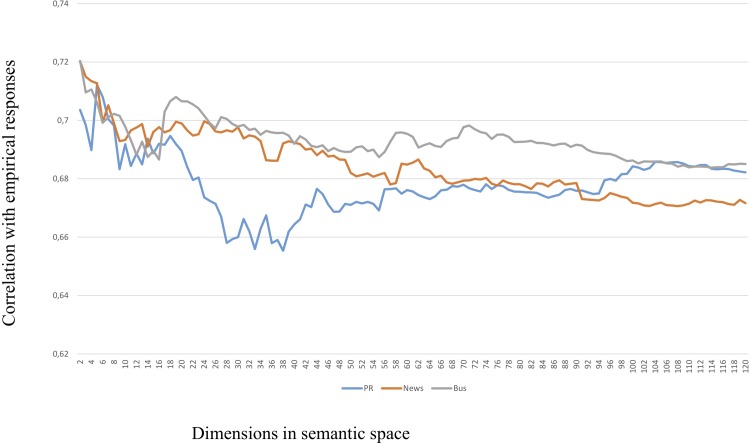
Correlations with motivation subset by semantic space and dimensions, 2–120 dimensions.

**Fig 5:**
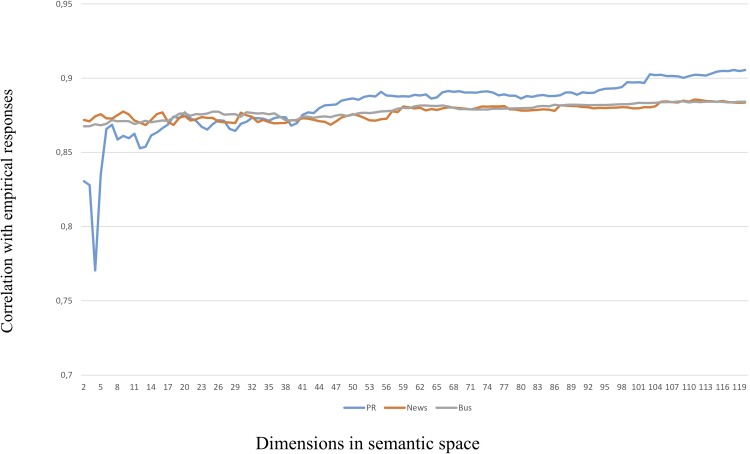
Correlations with outcomes subset by semantic space and dimensions, 2–120 dimensions. It turns out that the cosines produced by LSA using only very few dimensions are almost identical to the output of the MI algorithm, see Table 5.

The results were striking. Somewhat to our surprise, the LSA cosines for all three semantic spaces were most predictive of the empirical correlations in the very low range of dimensions (*k*) specified. This was unexpected as a higher number of dimensions is frequently found to make LSA perform better. This finding was true for the whole dataset, as well as for the three sub-categories leadership and motivation. Only in the case of organizational outcomes does it look as if better prediction is obtained by higher numbers of dimensions specified in the analysis. This phenomenon is illustrated in Figs 3, 4 and 5 below (in Figs 4 and 5, the range of dimensions is restricted to 120 to magnify the differences between semantic spaces).

**Table 5:** Correlations between LSA cosines and item response patterns predicted by MI values.

	MI predicted values	Residuals controlled for MI	MI predicted values	Residuals controlled for MI	MI predicted values	Residuals controlled for MI
Dimensions	Biz		News		PR	
2	.98	.04	.97	.04	.97	.06
3	.95	.02	.94	.03	.95	.06
4	.93	.01	.93	-.01	.93	.05
5	.92	-.01	.92	-.02	.90	.00
6	.91	-.03	.92	-.02	.91	-.01
7	.90	-.04	.91	-.03	.90	.00
8	.89	-.04	.90	-.04	.89	-.01
9	.89	-.04	.89	-.04	.88	-.02
10	.89	-.04	.88	-.03	.89	-.03
490	.83	-.07	.82	-.08	.84	-.07
491	.83	-.07	.82	-.08	.84	-.07
492	.83	-.07	.82	-.08	.84	-.07
493	.83	-.07	.82	-.08	.84	-.07
494	.83	-.07	.82	-.08	.84	-.07
495	.83	-.07	.82	-.08	.84	-.07
496	.83	-.07	.82	-.08	.84	-.07
497	.83	-.07	.82	-.08	.84	-.07
498	.83	-.07	.82	-.08	.84	-.07
499	.83	-.07	.82	-.08	.84	-.07
500	.83	-.07	.82	-.08	.84	-.07

In this way, the LSA cosines from low-dimensional spaces will attain the same predictive value as the “lexical” MI algorithm, but will also lose the additional predictive power of the cosines from higher dimensional spaces. Or, in other words, a higher number of dimensions will make LSA capture nuanced aspects of the meaning in the items at the loss of the main lexical bulk explained by the MI values. Therefore, the LSA cosines from different numbers of dimensional spaces will bring additional explanatory value. As can be seen from the Figs 3–5, any semantic space can outperform the others depending on which number of dimensions (*k*) chosen. Therefore, there is no simple “optimal” number of dimensions to choose from which the mutual performance of the semantic spaces can be decided.

We therefore chose a different procedure to test our hypotheses. It is possible to predict the empirical correlations in multiple regression applying a stepwise, forward entering regression procedure for each semantic space. In this procedure, the sets of cosines are entered and tested to find the optimal combination of semantic spaces to yield the best prediction of the empirical correlations. Since the low-dimensional semantic spaces are almost identical to the MI values, we first regressed the MI values on the empirical correlations and kept the residuals. The forward-entering procedure was then applied to predict the residuals (i.e., empirical correlations controlled for the MI values), again, for 2–500 dimensions of each semantic space. The results are displayed in Table 6.

**Table 6:** Empirical item correlations and controlled for MI, predicted in combinations of cosines from various dimensional spaces.

Predicting empirical correlations LSA only				Predicting stepwise with MI in the first block:						Predicting residuals of correlations after controlling for MI			
**Total datasets:**				**Total datasets:**						**Total datasets:**			
	Space	Max dim*	Adjusted R^2^		Space	MI R^2^	LSA R^2^	Increment	Max dim*		Space	Max dim*	Adjusted R^2^
	News	54	75		News	64	77	13	249		News	63	.348
	Biz	48	75		Biz	64	75	11	176		Biz	38	.243
	PR	76	77		PR	64	80	16	221		PR	34	.281
**Leadership**				**Leadership**						**Leadership**			
	Space	Max dim*	Adjusted R^2^		Space	MI R^2^	LSA R^2^	Increment	Max dim*		Space	Max dim*	Adjusted R^2^
	News	39	78		News	66	83	17	330		News	69	.475
	Biz	51	79		Biz	66	79	13	216		Biz	70	.426
	PR	47	79		PR	66	83	17	155		PR	47	.392
**Motive**				**Motive**						**Motive**			
	News	7	62		News	57	72	15	48		News	14	.449
	Biz	4	58		Biz	57	68	11	42		Biz	5	.325
	PR	1	51		PR	57	76	19	59		PR	6	.279
**Results**				**Results**						**Results**			
	News	20	90		News	76	90	14	58		News	31	.736
	Biz	11	85		Biz	76	87	11	44		Biz	16	.628
	PR	20	90		PR	76	89	13	68		PR	24	.738

* “Max dim” counts the maximum number of different dimensional resolutions included in the regression, not the number of dimensions.

Table 6 shows that combinations of LSA cosines generated from ranges of dimensions will predict the empirical correlations above the MI values and also above one single choice of dimensions. In this procedure, two of our three hypotheses gain support in that motives were best predicted through the News Cosines, and the outcomes were best predicted by the cosines generated from PR newswire (albeit with a minimal margin above the News cosines). The business press cosines failed to predict leadership in the present procedure, possibly attributable to the size of its semantic space. This semantic space is quite small compared to the others–only 40% the size of the PR semantic space and 52% of News, thus most susceptible to the effect of differences in text volumes.

## Discussion

The purpose of this study was to explore how the linguistic properties of different public media domains relate to survey responses in leadership research. Applying two different types of text analysis algorithms called latent semantic analysis (LSA) and MI, we projected OB survey items into three different semantic spaces: one from the business press (“BIZ”), one from *PR Newswire* (“PR”) and one from general newspapers (“NEWS”).

As hypothesised, our findings indicate that LSA cosines from three semantic spaces have different explanatory value for different groups of survey responses, depending on the type of items covered by the survey. Using the corpus-based algorithm MI as a control variable, a more “specialised” language understanding seemed to emerge from the three semantic spaces.

The semantic similarity indices were applied in a series of multiple linear regression models seeking to partial out their mutual levels of importance or dominance, a field that evokes problems of collinearity and suppressor variables[128]. One way of dealing with this problem is to decompose the regression weights into estimates of unique variance, common variance and dominance analysis [126]. These analyses basically supported our interpretation of the simple linear regressions, where all algorithm outputs are positively correlated with the observed correlation coefficients but may convey different types of information interpretable as suppressor variables[128].

Controlling for the lexical information supplied by the MI algorithm, our hypotheses were supported. The BIZ language did seem most fit to explain the leadership items, and the PR language did best in predicting answers related to organisational outcomes. The general NEWS language seemed most aligned with the everyday language in the lexical database, and was best fit to explain survey items measuring subjective motivational states.

These hypothesized effects were found using the commonly applied number of 300 dimensions (*k*) in the latent semantic analysis. As we conducted a thorough analysis of how the semantic spaces performed across a range of 2–500 dimensions (*k*), a somewhat more complex picture emerged. It turned out that low-dimensional LSA cosines were virtually equal to the values generated from the MI cosines. These can be seen as the crude, lexical estimates that explain the main bulk of empirical correlations, but still leave a proportion of variance unexplained. The cosines extracted from higher numbers of dimensions of semantic space seem to address finer aspects of language understanding, where each set of cosines may predict a unique part of the variance.

Contrary to our expectations, there were no simple solutions to determine the optimal number of dimensions that would decide the importance of each semantic space in explaining responses to sub-sets of items. Instead, we found that combinations of solutions within each semantic space would optimize the semantic space performance. This procedure allowed a replication of the results for motivation and outcomes, but not for leadership.

It is not easy to interpret these results, but they do seem to allow three conclusions: First of all, different semantic spaces do seem to pick up different aspects of language usage with differential predictive value in human responses to different constructs. Survey responses seem linked to differences in semantic constructions among communities of speakers. Secondly, the extraction of meaning from semantic spaces is very dependent on the number of dimensions (*k*) used. This does so far not seem to be a straightforward question of dimensional size because predictive performance of a semantic space can vary with no obvious relationship to the linear change in dimensions. Third, the size of the semantic space–measured in number texts, words and unique words, will influence the predictive power of cosines it produces.

These findings have several interesting implications related to OB research and organisation science. First, they emphasize the importance of understanding concepts in OB as emergent semantic constructs evolving as social constructions [4,5,129,130]. The business press, the PR community and the general reader of newspapers clearly develop language usage that differ in understanding of statements on results orientation, leadership topics or motivation. This is in accordance with the processes involved in sensemaking as described by Weick (e.g., [11,12,69,94]). Neither of the concepts explored here–leadership, motivation or results–have unequivocal and ubiquitous meanings [52,55,56,131–134]. Instead, they may be seen as fuzzy sets [135] with open indexicality [58,136]. To understand the meaning of these terms, speakers need to participate in the ongoing use of them in public contexts, where they take their meaning from debate and other types of exchange [130,137–139].

Second, our data indicate that researchers in this field are not uncovering objectively given structures, but instead working to construct their objects of interest–their constructs–through the use of items that elicit responses aligned with the purported qualities of the latent constructs. Scale construction is a tedious process [80] that needs to pick up the semantic habits of respondents. Research on uni-dimensional unfolding has previously shown that semantics are strongly related to the production of quantitative measurement data in surveys [95,96,140]. The correlation matrix or co-variance matrix of survey responses are usually the main input to statistic models of complex relationships among variables. Since linguistic information from different semantic spaces had differential relationships with the correlation matrices in our study, such semantic differences are likely to influence models established from survey data.

Third, our findings add more knowledge to the semantic theory of survey response (STSR). In these earlier studies, the semantic similarity indices were pooled into one group of predictors in multiple regression [31,33,34]. The justification for this was that the various algorithms were seen as imperfect compared to human language processing. Hence, a composite of several types of algorithms were used as a better approximation of human language parsing capability. The present study offers a rationale and an exploration for how and why different sets of semantic indices may vary in ways that may also be characteristic of human respondent subsamples. It also opens questions about how and why semantic values appear as suppressor variables when bundled in multiple regression [126,128], which requires more research on a detailed methodological level. We believe this provides an important advancement of STSR into modelling how semantics may differ between social domains such as professions, cultures or topics. In the previous studies on STSR, semantics could explain up to 86% of the variance in the correlation matrices of leadership measures. The findings of the present study suggest that additional variance may be explained if the semantic algorithms are sufficiently sensitive to the domain of items and the linguistic habits of the respondents.

This may also be useful to develop LSA in the future. According to Landauer et al. [99] one challenge in the development of LSA is to render the instrument sensitive to contexts. The present study adds evidence to this by showing how meaning represented in LSA will vary across domains, samples and topics.

Methodologically we have also encountered a challenge in comparing survey responses with semantic data. On a superficial level, the semantic indices are roughly comparable to the matrix of correlation coefficients. It is highly unlikely that this relationship is incidental. However, the relationship between semantics and attitude strength responses is complex and so far best described through unfolding theory [96]. The present study shows some of the complexity of working with standard regression models on network-based data. As we analytically tear off layers of information by controlling for other variables, parts of the basic relationships among the variables are still retained, probably through the multidimensional nature of networks. Our zero-order correlation matrix (Table 1) showed that the use of MI or the other LSA as control variables worked in the conventional way to create 0-correlations among the residuals of the equations. The unexpected finding is that these residuals still contained the same explanatory values (see Table 3). It is intriguing that the model statistics slowly approached curvilinear relationships, as if other dimensions of the semantic network come to bear on the relationships in the study. Future research will have to address this matter.

## Appendix A

### MI calculations

The MI sentence similarity measure is computed for two candidate sentences, S_1_ and S_2,_ as follows:

*Step 1*. *Identify part-of-speech (POS)*. The process begins with tokenisation and POS tagging of all the words in the survey item with their respective word classes (noun, verb, adverb, adjective and cardinal, which also plays a very important role in text understanding).

*Step 2*. *Calculate word similarity*. Each word in the sentence is measured against all the words from the other sentence to find the highest semantic similarity (maxSim) from six word-similarity metrics originally created to measure concept likeness rather than word likeness. The metrics are adapted here to compute word similarity by computing the shortest distance of given words’ synsets in the WordNet hierarchy. Word-word similarity is computed only on words from the same word class, which are either from noun or verb word classes because WordNet contains separate semantic trees for nouns and verbs. Thus, it is not possible to obtain similarity between nouns and verbs using WordNet distance. For other word classes such as adverb, adjective, cardinal and unknown words, whole-word matching is used instead. The word-word similarity measure is directional. It begins with each word in S1 being computed against each word in S2, and then, vice versa.

*Step 3*. *Calculating sentence similarity*. Once the highest semantic similarity (maxSim) for each word in the sentences is computed, it is normalised by applying Inverse Document Frequency (IDF) to the British National Corpus to weight rare and common terms. The normalised scores are then summed up for a sentence similarity score, SimMI, as follows:

$${\text{Sim}}_{\text{MI}}\left({\text{S}}_{1},{\text{S}}_{2}\right)=\frac{1}{2}\times \frac{\sum_{\left(\text{w}\in {\text{S}}_{1}\right)}\text{maxSim}\left(\text{w},{\text{S}}_{2}\right)\times \text{IDF}\left(\text{w}\right)}{\sum_{\left(\text{w}\in {\text{S}}_{1}\right)}\text{IDF}\left(\text{w}\right)}+\frac{\sum_{\left(\text{w}\in {\text{S}}_{2}\right)}\text{maxSim}\left(\text{w},{\text{S}}_{1}\right)\times \text{IDF}\left(\text{w}\right)}{\sum_{\left(\text{w}\in {\text{S}}_{2}\right)}\text{IDF}\left(\text{w}\right)},$$

where maxSim(w, S2) is the score of the most similar word in S2 to w and IDF(w) is the IDF of word w.

### LSA calculations

Only an overview is given of the four core steps of the LSA process because of the careful treatment of LSA elsewhere, including by one of the authors (Larsen & Monarchi, 2004).

*Step 1*. *Preparing term-document matrix*. LSA starts by creating a term-document matrix, A, containing a weighted count of how many times a word, i, appears inside a document, j. The weighting method employed here, log-entropy, has been found generally to outperform other LSA weighting schemes (Dumais, 1991; Nakov et al., 2001).

*Step 2*. *Creating semantic space*. After appropriate preparation (weighting, normalisation, etc.), this matrix is decomposed using Singular Value Decomposition, a mathematical algorithm similar to a factor analysis, with the result being a semantic space of a given dimension represented as three matrices: U, a term-by-dimension matrix representing words; S, a singular value matrix; and V, a document-by-dimension matrix representing documents. The equation can be written as:

$$A=USV^{T},$$

U and V are orthogonal matrices whereas S is a diagonal matrix with main diagonal entries sorted in decreasing order. In practice, A could be approximated with A_K_ by preserving the first k singular values and the corresponding first k columns in U and V. The approximation can be written as:

$${\text{A}}_{\text{k}}\approx {\text{U}}_{\text{k}}{\text{S}}_{\text{k}}{\text{V}}_{\text{k}}^{\text{T}},$$

where U_*k*_ is a term-by-k matrix, S_*k*_ is a k-by-k matrix and *V*_*k*_ is a document-by-k matrix. This approximation estimates A with minimal error and also translates the term-by-document matrix into a correlated semantic space. Thus, each row vector of U_*k*_S_*k*_ represents a word in the semantic space and has k columns which give the vector of the word in the semantic space. Likewise, each row of *V*_*k*_*S*_*k*_ represents a document vector that correlates topics in the semantic space. By preserving the first k diagonal elements in S, the low-rank approximation has produced the mutual constraints among words in different documents.

*Step 3*. *Projecting items into the semantic space*. Given the query ***q***, which is a survey item, query vector ***q*** is obtained through an aggregation of word vectors relevant to the item. In our research, every item is projected into the semantic space as a query vector, $\vec{q}$, and that vector is saved as ***q***_*n*_ for future item-item analysis, where *n* is the total number of items.

*Step 4*. *Calculating the similarity of items*. To find similar items to $\vec{q}$, the query vector is then compared against all the items stored inside the semantic space, $\vec{q}$_n_, using the cosine similarity measurement, where n is the total number of stored items:

$$Similar\left(q\right)=\sum_{n}^{i}\cos(\vec{q},{\vec{q}}_{n}).$$

Stemming: The items were pre-processed in the same way as the underlying semantic space. This concretely means that the Porter [141] stemming algorithm was applied.

## Supporting information

S1 Table Complete dataset as used in the article. All variables necessary to replicate our results with two exceptions: a) The MLQ items are copyright protected and only included with item numbers and semantic values, b) the replications with 2–500 dimensions are too extensive to be replicated here.
(XLSX)
